# Cardiorespiratory responses to exercise related to post-stroke fatigue severity

**DOI:** 10.1038/s41598-021-92127-w

**Published:** 2021-06-17

**Authors:** Kazuaki Oyake, Yasuto Baba, Yuki Suda, Jun Murayama, Ayumi Mochida, Yuki Ito, Honoka Abe, Kunitsugu Kondo, Yohei Otaka, Kimito Momose

**Affiliations:** 1 Department of Physical Therapy, School of Health Sciences, Shinshu University, 3-1-1 Asahi, Matsumoto, Nagano 390-8621 Japan; 2 Department of Rehabilitation Medicine, Tokyo Bay Rehabilitation Hospital, Chiba, Japan; 3 Department of Rehabilitation Medicine I, School of Medicine, Fujita Health University, Aichi, Japan

**Keywords:** Health care, Rehabilitation, Neurology

## Abstract

Physical deconditioning after stroke may induce post-stroke fatigue. However, research on this association is limited. Our primary objective was to investigate the associations of post-stroke fatigue severity with oxygen uptake ($\dot{\mathrm{V}}$O_2_) at peak exercise and the time constant of $\dot{\mathrm{V}}$O_2_ kinetics (τ$\dot{\mathrm{V}}$O_2_) at exercise onset. The secondary objective was to examine the associations between fatigue and cardiorespiratory variables potentially affecting $\dot{\mathrm{V}}$O_2_ during exercise. Twenty-three inpatients from a subacute rehabilitation ward were enrolled in this study. The median (interquartile range) Fatigue Severity Scale (FSS) score, as a measure of fatigue, was 32 (range 27–42) points. The FSS score was not associated with $\dot{\mathrm{V}}$O_2_ at peak exercise during a symptom-limited graded exercise test (rho = − 0.264; p = 0.224), whereas it was significantly associated with τ$\dot{\mathrm{V}}$O_2_ during a submaximal constant-load exercise test (rho = 0.530; p = 0.009). A higher FSS score also significantly correlated with a longer time constant of cardiac output (CO) kinetics (rho = 0.476; p = 0.022). Our findings suggest that severe post-stroke fatigue is associated with delayed increases in $\dot{\mathrm{V}}$O_2_ and CO at the onset of exercise. Our findings can contribute to the development of an appropriate rehabilitation programme for individuals with post-stroke fatigue.

## Introduction

Post-stroke fatigue is defined as ‘a subjective lack of physical and/or mental energy that is perceived by the individual to interfere with usual or desired activities’^1,2^. A systematic review reported that the prevalence of post-stroke fatigue ranged between 25 and 85%^2^. Post-stroke fatigue is associated with various factors, such as depressive symptoms and functional disability^1^, and individuals with post-stroke fatigue are reported to have poor recovery in terms of activities of daily living^3,4^, a lower rate of returning to work^5^, reduced health-related quality of life^6^, and increased mortality^4,7^. The underlying pathophysiology of post-stroke fatigue is not completely understood^1^. Although exercise training can improve fitness, balance, mobility, and activities of daily living in individuals with stroke^8^, there is insufficient evidence regarding the effectiveness of rehabilitative exercise programmes for improving post-stroke fatigue^1,9^.

It has been suggested that post-stroke fatigue is triggered through physical deconditioning, which may lead to the avoidance of physical activities and further deconditioning^10^. Oxygen uptake ($\dot{\mathrm{V}}$O_2_) at peak exercise, measured during a symptom-limited graded exercise test, is widely accepted as an indicator of cardiorespiratory capacity in individuals with stroke^8,11,12^. $\dot{\mathrm{V}}$O_2_ at peak exercise in individuals with stroke is 26–87% of that in healthy age- and sex-matched individuals^11^. Moreover, the assessment of $\dot{\mathrm{V}}$O_2_ kinetics at the onset of submaximal exercise has also been shown to provide objective information on the cardiorespiratory fitness of individuals with stroke^13,14^. Transient measurements of $\dot{\mathrm{V}}$O_2_ in a constant-load exercise at an intensity below the ventilatory threshold are classified into three phases. Time constant of $\dot{\mathrm{V}}$O_2_ in phase II (τ$\dot{\mathrm{V}}$O_2_) has often been used to assess $\dot{\mathrm{V}}$O_2_ kinetics at the onset of exercise, which reflects the ability of the cardiorespiratory system to adapt from rest to a new steady-state during submaximal exercise^15^. A longer τ$\dot{\mathrm{V}}$O_2_ is associated with poorer health status, ageing, and a sedentary lifestyle^15,16^. Tomczak et al.^13^ reported that τ$\dot{\mathrm{V}}$O_2_ was greater in individuals with stroke than in age-, sex-, and activity-matched healthy adults. However, there is limited evidence concerning the associations between post-stroke fatigue and these cardiorespiratory fitness variables; therefore, this study aimed to investigate these associations.

The variables involved in post-stroke fatigue may differ based on the severity/stage of stroke. Wu et al.^17^ proposed a conceptual model of post-stroke fatigue, wherein biological factors are expected to trigger fatigue at the early stage after stroke (usually within the first three months after stroke), whereas fatigue at the later stage after stroke (usually > 1 year after stroke) is attributed to psychological and behavioural factors. A cross-sectional study on individuals with chronic stroke (4.1 ± 3.5 years post-stroke) reported that post-stroke fatigue was associated with depressive symptoms but not with $\dot{\mathrm{V}}$O_2_ at peak exercise^18^. However, the association between post-stroke fatigue at the early stage after stroke and cardiorespiratory fitness has not been reported. The primary objective of this study was to examine the associations of the severity of post-stroke fatigue with $\dot{\mathrm{V}}$O_2_ at peak exercise obtained during a symptom-limited graded exercise test and τ $\dot{\mathrm{V}}$O_2_ at the onset of exercise measured during a submaximal constant-load exercise test in inpatients at a subacute rehabilitation ward. In previous studies, τ$\dot{\mathrm{V}}$O_2_ has been reported to be more sensitive to changes in the levels of physical activity compared with $\dot{\mathrm{V}}$O_2_ at peak exercise^19–21^. Moreover, post-stroke fatigue has been found to be related to lower levels of physical activity^22^. Based on these findings, we hypothesised that the severity of post-stroke fatigue would likely be more strongly associated with τ$\dot{\mathrm{V}}$O_2_ than $\dot{\mathrm{V}}$O_2_ at peak exercise.

In addition, respiratory and cardiac function impairment in relation to supplying oxygen and an inability of skeletal muscles to extract oxygen may limit the increase in $\dot{\mathrm{V}}$O_2_ during exercise in individuals with stroke^13,23,24^. Therefore, our secondary objective was to identify associations between post-stroke fatigue and cardiorespiratory variables potentially affecting $\dot{\mathrm{V}}$O_2_ during exercise, such as the oxygen uptake efficiency slope (OUES), cardiac output (CO), and arterial-venous oxygen difference (AVO_2_diff). Ventilatory efficiency and muscle oxygen extraction, measured using OUES and AVO_2_diff, respectively, have been reported to be lower in individuals with stroke than in healthy adults^25,26^. Therefore, we hypothesised that impairment of these variables would also be associated with post-stroke fatigue. Elucidating cardiorespiratory factors associated with post-stroke fatigue could contribute to the development of an appropriate rehabilitation programme for individuals with post-stroke fatigue.

## Results

### Participants

**Figure 1:**
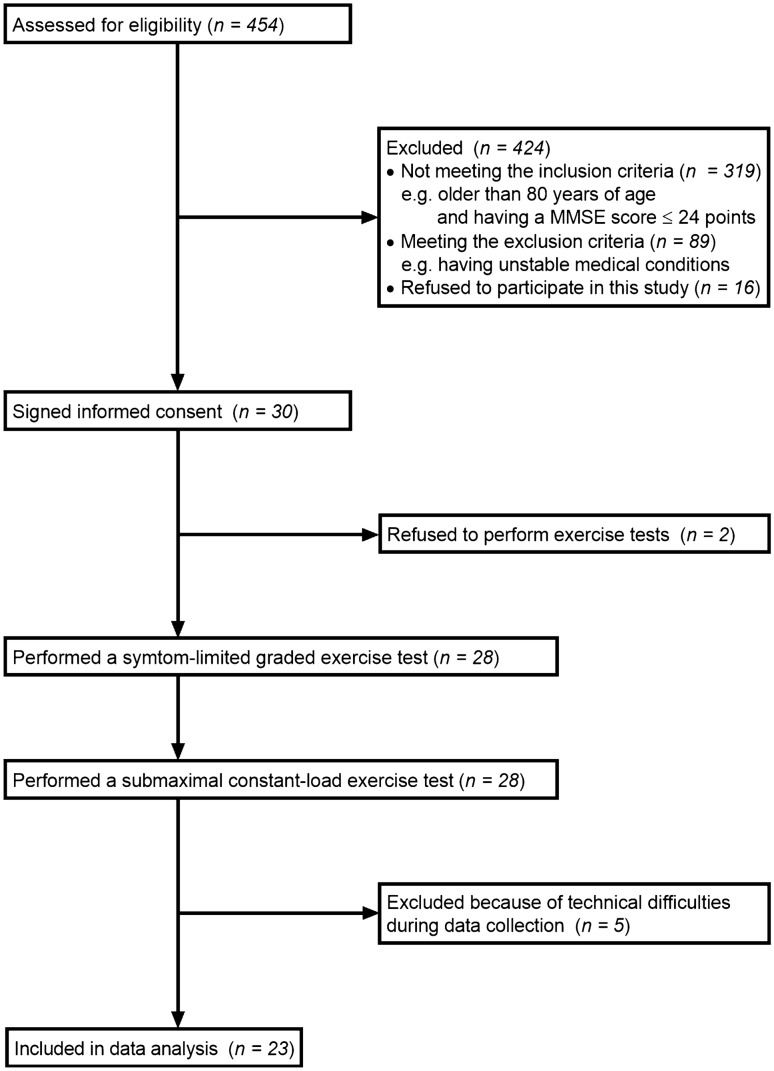
Flow diagram of study participants. *MMSE* Mini-Mental State Examination.

A flow chart of participants enrolled in the study is shown in Fig. 1. Thirty individuals with stroke provided informed consent. However, two participants declined to perform exercise tests. Furthermore, in five of 28 participants who performed the submaximal constant-load exercise test, cardiorespiratory data during the test could not be measured because of technical difficulties. Consequently, data concerning 23 participants were included in the analysis. Although all participants were recruited from a subacute rehabilitation ward, five participants were in the chronic phase of stroke recovery (≥ 3 months after stroke)^27^. Table 1 shows the participants’ characteristics.

**Table 1:** Associations between the Fatigue Severity Scale score and participants’ characteristics.

Variable	Value	rho	p value	W value	p value
Age, years	59.8 ± 10.0	− 0.007	0.973	NA	NA
Sex, male/female	17/6	NA	NA	67.0	0.277
Height, m	1.66 ± 0.07	− 0.082	0.712	NA	NA
Weight, kg	62.0 ± 9.0	0.210	0.337	NA	NA
Body mass index, kg m^−2^	22.4 ± 3.3	0.173	0.430	NA	NA
Type of stroke, ischaemic/haemorrhagic	12/11	NA	NA	81.0	0.371
Side of motor paresis, right/left	11/12	NA	NA	63.0	0.877
Time since stroke, days	69.7 ± 30.2	− 0.069	0.753	NA	NA
**Comorbidities**					
Hypertension	11	NA	NA	69.5	0.853
Diabetes mellitus	5	NA	NA	34.5	0.455
FSS score, points	32 (27, 42)	NA	NA	NA	NA
GDS score, points	5 (2, 6)	NA	NA	NA	NA
Depressive symptoms (GDS score ≥ 5)	13	NA	NA	54.5	0.534
Mini-Mental State Examination score, points	28 (26, 30)	0.085	0.701	NA	NA
Stroke Impairment Assessment Set motor score, points	19 (12, 25)	− 0.237	0.276	NA	NA
Functional Independence Measure motor score, points	77 (70, 89)	− 0.140	0.524	NA	NA
Functional Independence Measure cognitive score, points	30 (28, 35)	0.029	0.895	NA	NA

Values are presented as mean ± standard deviation, median (interquartile range), or number.

*rho* Spearman’s rank correlation coefficient, *FSS* Fatigue Severity Scale, *GDS* Geriatric Depression Scale, *NA* not available.

### Exercise testing

No significant adverse events occurred during or after the exercise tests. All participants had to stop the symptom-limited graded exercise test due to their inability to maintain a cycling cadence of > 40 rpm. Concerning each of the three criteria for reaching the maximal effort, 21 (91.3%) participants had an increase in $\dot{\mathrm{V}}$O_2_ of < 150 mL min^−1^ for > 1 min despite an increased work rate, six (26.1%) achieved a respiratory exchange ratio of > 1.10, and 11 (47.8%) reached 85% of the age-predicted maximal heart rate. The ventilatory threshold was determined for all participants.

Regarding cardiorespiratory variables measured during the submaximal constant-load exercise test, the mean ± standard deviation (SD) coefficients of determination of the kinetics of $\dot{\mathrm{V}}$O_2_, CO, AVO_2_diff, and minute ventilation ($\dot{\mathrm{V}}$E) were 0.99 ± 0.01, 0.96 ± 0.02, 0.93 ± 0.03, and 0.98 ± 0.01, respectively. In addition, the mean ± SD ratio of the time constant of CO (τCO) to τ$\dot{\mathrm{V}}$O_2_ was 1.19 ± 0.56. In 15 of 23 (65.2%) participants, the ratio of τCO to τ$\dot{\mathrm{V}}$O_2_ was > 1.00.

Measurement values obtained during the symptom-limited graded and submaximal constant-load exercise tests are shown in Table 2.

**Table 2:** Correlations between the Fatigue Severity Scale score and cardiorespiratory variables during a symptom-limited graded exercise test and a submaximal constant-load exercise test.

Variable	Value	Correlation analysis	
		rho (95% CI)	p value
**At peak exercise during graded exercise**			
$\dot{\mathrm{V}}$O_2_, mL kg^−1^ min^−1^	18.0 ± 4.2	− 0.264 (− 0.618, 0.179)	0.224
CO, L min^−1^	9.2 ± 1.4	− 0.017 (− 0.437, 0.409)	0.939
AVO_2_diff, mL 100 mL^−1^	12.2 ± 2.5	− 0.106 (− 0.506, 0.332)	0.632
$\dot{\mathrm{V}}$E, L min^−1^	43.4 ± 14.7	− 0.011 (− 0.432, 0.414)	0.959
Respiratory exchange ratio	0.99 ± 0.12	0.104 (− 0.334, 0.504)	0.638
Oxygen uptake efficiency slope, mL min^−1^·log(L min^−1^)^−1^	1,354 ± 355	− 0.401 (− 0.705, 0.026)	0.058
**At ventilatory threshold during graded exercise**			
$\dot{\mathrm{V}}$O_2_, mL kg^−1^ min^−1^	14.2 ± 2.9	− 0.294 (− 0.637, 0.148)	0.174
CO, L min^−1^	7.8 ± 1.1	− 0.105 (− 0.505, 0.333)	0.634
AVO_2_diff, mL 100 mL^−1^	11.2 ± 1.9	− 0.078 (− 0.485, 0.357)	0.724
$\dot{\mathrm{V}}$E, L min^−1^	24.1 ± 6.9	− 0.019 (− 0.438, 0.407)	0.932
Respiratory exchange ratio	0.74 ± 0.08	0.021 (− 0.405, 0.440)	0.923
**At the onset of constant-load exercise**			
τ$\dot{\mathrm{V}}$O_2_, s	38.6 ± 10.1	0.530 (0.138, 0.778)	0.009
τCO, s	45.3 ± 22.5	0.476 (0.067, 0.749)	0.022
τAVO_2_diff, s	26.7 ± 7.7	0.215 (− 0.228, 0.585)	0.324
τ$\dot{\mathrm{V}}$E, s	70.0 ± 15.1	0.234 (− 0.210, 0.598)	0.283

Values are presented as mean ± standard deviation.

*Rho* Spearman’s rank correlation coefficient, $\dot{V}$*O*_*2*_ oxygen uptake, *CO* cardiac output, *AVO*_*2*_*diff* arterial-venous oxygen difference, $\dot{V}$*E* minute ventilation, *τ* the time constant.

### Correlations between the Fatigue Severity Scale score and cardiorespiratory variables measured during the symptom-limited graded and submaximal constant-load exercise tests (Table 2)

The median (interquartile range) Fatigue Severity Scale (FSS) score was 32 (range 27–42). The mean ± SD $\dot{\mathrm{V}}$O_2_ values at peak exercise and τ$\dot{\mathrm{V}}$O_2_ at the onset of exercise were 18.0 ± 4.2 mL kg^−1^ min^−1^ and 38.6 ± 10.1 s, respectively.

**Figure 2:**
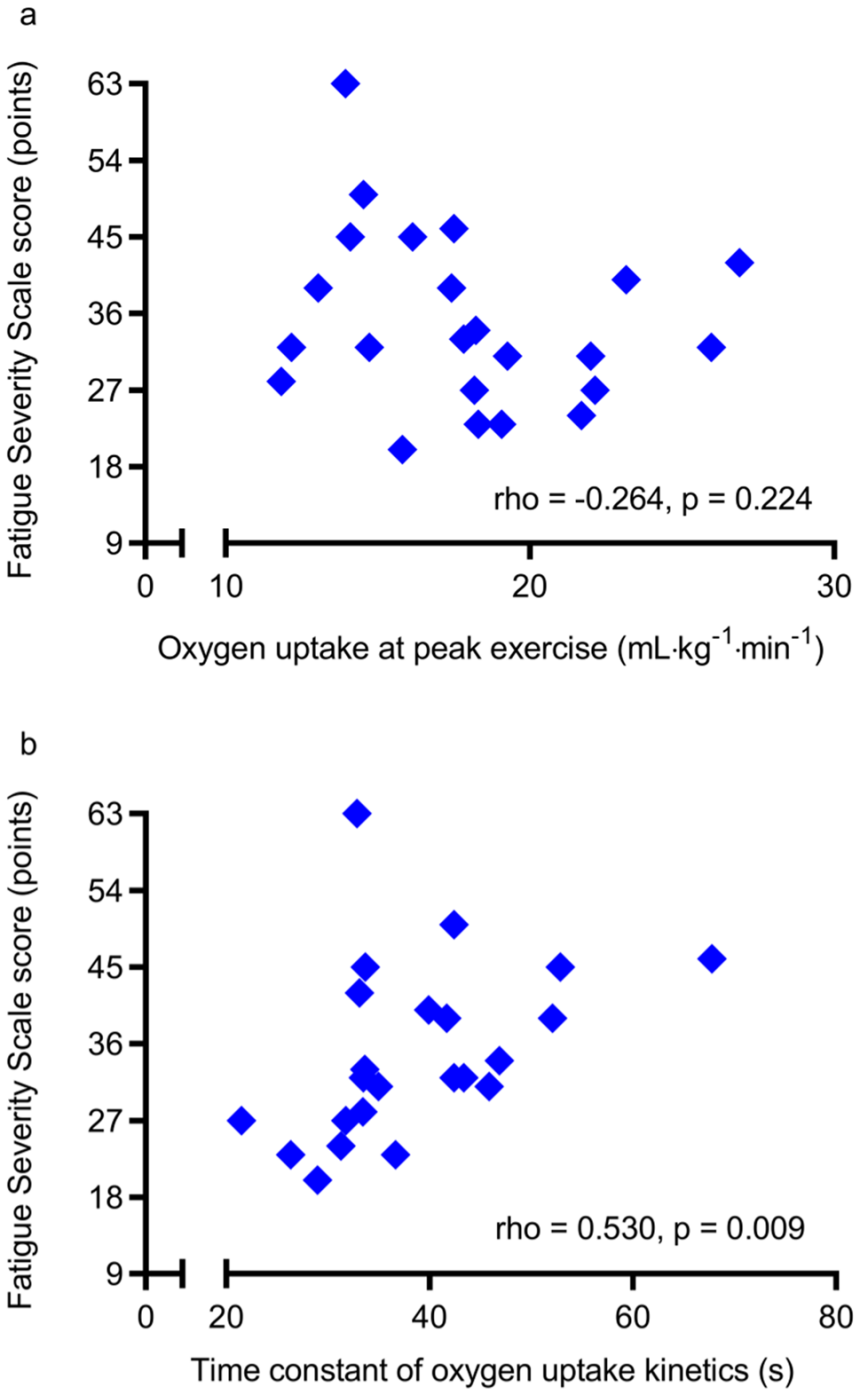
Correlations of the Fatigue Severity Scale score with (**a**) oxygen uptake at peak exercise and (**b**) the time constant of oxygen uptake kinetics.

**Figure 3:**
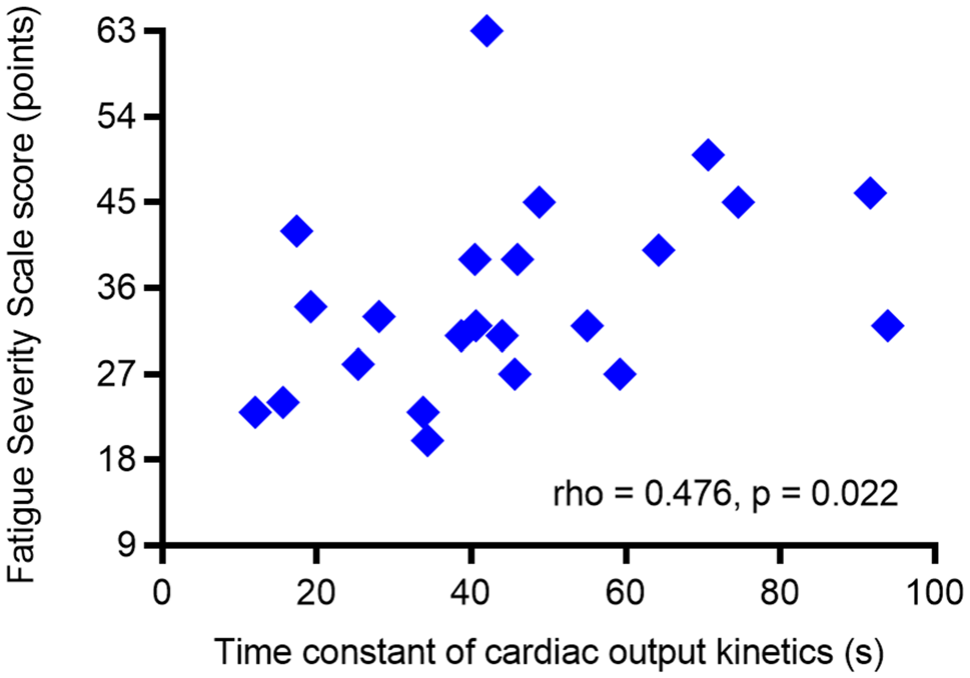
Correlation between the Fatigue Severity Scale score and the time constant of cardiac output kinetics.

The FSS score was not significantly correlated with $\dot{\mathrm{V}}$O_2_ at peak exercise (rho = − 0.264; p = 0.224, Fig. 2a), whereas a higher FSS score significantly correlated with a longer τ$\dot{\mathrm{V}}$O_2_ (rho = 0.530; p = 0.009, Fig. 2b). Although the FSS score was not associated with the other cardiorespiratory variables at peak exercise and at the ventilatory threshold measured during the symptom-limited exercise test, a higher FSS score was significantly associated with a longer τCO (rho = 0.476; p = 0.022, Fig. 3) during the submaximal constant-load exercise test. Changes in $\dot{\mathrm{V}}$O_2_ and CO at the onset of exercise in representative participants with different fatigue levels (low and high) are shown in Supplementary Figs. S1 and S2, respectively, online.

### The association between the FSS score and τ$\dot{\mathbf{V}}$O_2_ after adjusting for participants’ characteristics

The FSS score was not found to be significantly associated with participants’ characteristics, including age, sex, height, weight, body mass index, type of stroke, side of motor paresis, time since stroke, presence of hypertension and diabetes mellitus, Mini-Mental State Examination (MMSE) score, presence of depressive symptoms, Stroke Impairment Assessment Set motor score, and Functional Independence Measure score in the motor and cognition items (p > 0.05, Table 1). τ$\dot{\mathrm{V}}$O_2_ also showed no significant associations with participants’ characteristics (p > 0.05, Table 3). Nevertheless, we additionally performed multiple regression analyses to confirm whether the association between the FSS score and τ$\dot{\mathrm{V}}$O_2_ remained significant, even when adjusting for the logically confounding variables such as age, sex, type of stroke, time since stroke, presence of depressive symptoms, and Functional Independence Measure motor score^1,15,28^. These variables were entered into the regression model one by one. The regression models were significant when adjusting for sex or type of stroke, and the association between the FSS score and τ$\dot{\mathrm{V}}$O_2_ remained significant even after controlling for sex (F (2, 20) = 4.597; p = 0.023) or type of stroke (F (2, 20) = 3.754; p = 0.041) (Supplementary Table S1).

**Table 3:** Associations between the time constant of oxygen uptake and participants’ characteristics.

Variable	τ$\dot{\mathrm{V}}$O_2_	
	Correlation coefficient (p value)	t value (p value)
Age	r = 0.272 (0.209)	NA
Sex	NA	− 0.160 (0.875)
Height	r = − 0.096 (0.662)	NA
Weight	r = 0.066 (0.766)	NA
Body mass index	r = − 0.085 (0.699)	NA
Type of stroke	NA	− 1.085 (0.290)
Side of motor paresis	NA	− 0.657 (0.518)
Time since stroke	r = − 0.007 (0.973)	NA
**Comorbidities**		
Hypertension	NA	− 1.377 (0.183)
Diabetes mellitus	NA	− 1.665 (0.111)
Depressive symptoms (GDS score ≥ 5)	NA	0.083 (0.935)
Mini-Mental State Examination score	rho = 0.072 (0.744)	NA
Stroke Impairment Assessment Set motor score	rho = − 0.113 (0.608)	NA
Functional Independence Measure motor score	rho = 0.017 (0.940)	NA
Functional Independence Measure cognitive score	rho = − 0.236 (0.278)	NA

*τ*$\dot{V}$*O*_*2*_ time constant of oxygen uptake kinetics, *r* Pearson’s product-moment correlation coefficient, *rho* Spearman’s rank correlation coefficient, *GDS* Geriatric Depression Scale, *NA* not available.

## Discussion

The primary objective of this study was to examine associations of the severity of post-stroke fatigue with $\dot{\mathrm{V}}$O_2_ at peak exercise obtained during a symptom-limited graded exercise test and τ$\dot{\mathrm{V}}$O_2_ at the onset of exercise measured during a submaximal constant-load exercise test in inpatients at a subacute rehabilitation ward. The results of post-stroke fatigue assessment in this study are similar to those of previous studies^29,30^. This study showed that a higher FSS score was associated with a longer τ$\dot{\mathrm{V}}$O_2_ at the onset of exercise, but not with $\dot{\mathrm{V}}$O_2_ at peak exercise. Our secondary objective was to identify the associations between post-stroke fatigue and cardiorespiratory variables potentially affecting $\dot{\mathrm{V}}$O_2_ during exercise, such as the OUES, CO, and AVO_2_diff. Our findings showed that a higher FSS score was associated with a longer τCO at the onset of exercise, which suggests that the severity of post-stroke fatigue is related to delayed increases in $\dot{\mathrm{V}}$O_2_ and CO at the onset of exercise. Additionally, the FSS score and τ$\dot{\mathrm{V}}$O_2_ had no significant associations with age, sex, height, weight, body mass index, type of stroke, side of motor paresis, time since stroke, presence of hypertension and diabetes mellitus, MMSE score, presence of depressive symptoms, Stroke Impairment Assessment Set motor score, and Functional Independence Measure score in the motor and cognition items. Moreover, the association between the FSS score and τ$\dot{\mathrm{V}}$O_2_ remained significant, even when we adjusted for sex or type of stroke. Therefore, the confounding effects of the participants’ characteristics on the association between the FSS score and τ$\dot{\mathrm{V}}$O_2_ appear to be limited.

Individuals with post-stroke fatigue lack the energy necessary to perform activities, are more easily tired due to activity, experience unpredictable and unexplainable feelings of fatigue, and have increased stress sensitivity and an increased need for longer sleep durations, naps, or rest^31^. Thus, it is plausible that post-stroke fatigue might be associated with decreased cardiorespiratory fitness and reduced physical activity^10,32^. However, no statistically significant correlation between the FSS score and $\dot{\mathrm{V}}$O_2_ at peak exercise has been shown in individuals with chronic stroke^18^. To our knowledge, this study is the first to investigate association between fatigue at an early post-stroke stage and cardiorespiratory fitness. Our finding that the association between the FSS score and $\dot{\mathrm{V}}$O_2_ at peak exercise was not significant was consistent with that of a previous study involving patients with chronic stroke^18^, whereas the association between the FSS score and τ$\dot{\mathrm{V}}$O_2_ measured during the submaximal constant-load exercise test was significant. We observed that the mean coefficient of determination of cardiorespiratory kinetics was > 0.85, which indicated that the fitting procedures were acceptable^33^. The mean value of τ$\dot{\mathrm{V}}$O_2_ in this study was similar to the value obtained in a previous study that found that individuals with stroke had a longer τ$\dot{\mathrm{V}}$ O_2_ than age-, sex-, and activity-matched healthy adults^13^. In healthy adults, the acceleration of τ$\dot{\mathrm{V}}$O_2_ has been reported to occur in the early period of endurance training, and $\dot{\mathrm{V}}$O_2_ at peak exercise subsequently increases^19,21^. Additionally, a previous study reported that τ$\dot{\mathrm{V}}$O_2_ was shorter in a recreationally active group than in an inactive group; however, there was no significant difference in $\dot{\mathrm{V}}$O_2_ at peak exercise between the two groups^16^. Based on these findings, τ$\dot{\mathrm{V}}$O_2_ may be more sensitive to changes in the levels of physical activity compared with $\dot{\mathrm{V}}$O_2_ at peak exercise. Therefore, the associations of the FSS score with τ$\dot{\mathrm{V}}$O_2_ and $\dot{\mathrm{V}}$O_2_ at peak exercise observed in this study suggest that τ$\dot{\mathrm{V}}$O_2_ more sensitively reflects lower levels of physical activity in individuals with post-stroke fatigue than $\dot{\mathrm{V}}$O_2_ at peak exercise.

We found that the mean ratio of τCO to τ$\dot{\mathrm{V}}$O_2_ was > 1.00, indicating that oxygen delivery did not exceed the metabolic demand during exercise onset and that $\dot{\mathrm{V}}$O_2_ kinetics at exercise onset were limited owing to a delayed increase in CO^34^. However, it is unclear why participants with severe post-stroke fatigue showed a delayed increase in CO at the onset of exercise. A prompt increase in CO at the onset of exercise is compatible with the notion of immediate vagal withdrawal. Capelli et al.^35^ reported that the increase in CO at the onset of exercise slowed after than before prolonged bed rest in healthy adults, because of a decrease in vagal activity at rest and elimination of vagal withdrawal during exercise. In addition, reduced cardiac mass and function, plasma volume, and venous return after prolonged bed rest^36–39^ may also negatively affect the increase in CO during exercise onset. Furthermore, given that post-stroke fatigue is associated with low physical activity^22^, an inactive lifestyle may act as a confounder in relation to severe fatigue and delayed increases in $\dot{\mathrm{V}}$O_2_ and CO at the onset of exercise. An assessment of physical activity is needed in future studies to determine the reasons for these associations of the FSS score with τ$\dot{\mathrm{V}}$O_2_ and τCO observed in this study.

Our findings suggest that individuals with severe post-stroke fatigue need to improve τ$\dot{\mathrm{V}}$O_2_ at the onset of exercise. Previous studies have shown that aerobic exercise training was effective in the improvement of τ$\dot{\mathrm{V}}$O_2_ in older individuals^20,40,41^. A randomised controlled trial reported that a combination of cognitive-behavioural therapy and graded activity training was more effective than cognitive-behavioural therapy alone in treating post-stroke fatigue^42^. Although post-stroke fatigue has a negative effect on recovery of activities of daily living^3,4^, one systematic review reported that aerobic exercise can improve functional ability in individuals with stroke^8^. Furthermore, in the subacute phase of stroke recovery, several studies have shown the effectiveness of exercise in improving health outcomes, including cardiovascular, functional, and mobility outcomes, after stroke^43,44^. Therefore, rehabilitative exercise programmes may be beneficial for individuals with post-stroke fatigue.

The use of exercise testing for clinical assessment and exercise prescription is limited in stroke rehabilitation settings^45,46^, thus limiting the clinical applicability of our findings. A lack of exercise equipment, time, space, and support staff have also been reported as barriers to exercise testing^45^. Moreover, cardiac, cognitive, functional, and physical impairments in individuals with stroke may make it difficult to perform exercise testing safely^45^. More specific clinical guidelines for post-stroke exercise testing, educational training associated with exercise testing, and greater collaboration between stroke and cardiac rehabilitation teams could help to implement exercise testing more effectively in stroke rehabilitation settings^45,46^.

This study had some limitations. First, the sample size was relatively small because we only calculated the sample size required for a bivariate correlation analysis. Post-stroke fatigue has been found to be associated with older age, female sex, depressive symptoms, and functional disability^1^. Changes in the brain after a stroke may also affect post-stroke fatigue and cardiorespiratory control during exercise^1,28^. Even though our participants were recruited from a subacute rehabilitation ward, five participants were in the chronic phase of stroke recovery. In addition, 12 participants with ischaemic stroke and 11 with haemorrhagic stroke were included in this study. Functional recovery differs between individuals with ischaemic and haemorrhagic strokes. Stroke severity is higher in haemorrhagic stroke than in ischaemic stroke, while individuals with haemorrhagic stroke have been shown to have a higher therapeutic response to rehabilitation than those with ischaemic stroke^47,48^. However, our study findings indicated that these variables were not associated with the FSS score and τ$\dot{\mathrm{V}}$O_2_. Further studies using multivariate analysis and a sufficiently large sample size are warranted to confirm the robustness of our findings.

Second, most participants were in the subacute phase of stroke recovery. Because fatigue at a later stage after stroke may be associated more with psychological and behavioural factors than with biological factors^17^, generalising our findings to individuals in the later stage after stroke should be made with caution.

Third, many individuals with stroke (n = 424) were excluded from the study. Many of them were excluded due to being > 80 years of age, having an MMSE score of ≤ 24 points^49^, and/or having unstable medical conditions, as shown in Fig. 1. This may limit the generalisability of our findings in relation to individuals with these conditions.

Finally, because this study used a cross-sectional observational design, the cardiorespiratory variables associated with temporal changes in post-stroke fatigue could not be examined. Thus, further longitudinal studies are needed to investigate the temporal association between post-stroke fatigue and cardiorespiratory fitness variables.

In summary, a higher FSS score statistically significantly correlated with longer τ$\dot{\mathrm{V}}$O_2_ at the onset of exercise measured during a submaximal constant-load exercise test, but not with $\dot{\mathrm{V}}$O_2_ at peak exercise obtained during a symptom-limited graded exercise test. In addition, a higher FSS score was associated with a longer τCO at the onset of exercise. These results suggest that severe post-stroke fatigue is related to delayed increases in $\dot{\mathrm{V}}$O_2_ and CO at the onset of exercise. Collectively, our findings can contribute to the development of an appropriate rehabilitation programme for individuals with post-stroke fatigue.

## Methods

### Study design

This cross-sectional study’s protocol was approved by the appropriate ethics committees of Tokyo Bay Rehabilitation Hospital (approval number, 172-2) and Shinshu University (approval number: 3813), and conducted according to the Declaration of Helsinki of 1964 as revised in 2013. All participants provided written informed consent before enrolment.

### Participants

Participants were recruited from a subacute rehabilitation ward between November 2017 and March 2020. Inclusion criteria comprised the following: age 40–80 years, within 180 days of the initial stroke, an ability to maintain a target cadence of 50 rpm during exercise, and an MMSE score of > 24^49^. Exclusion criteria comprised the following: limited range of motion and/or pain that could affect the exercise test; unstable medical conditions, such as unstable angina, uncontrolled hypertension, or tachycardia; the use of beta-blocker medication; and any comorbid neurological disorders. Demographic and clinical data, such as age and type of stroke, were obtained from patient medical records.

### Procedure

Data collection was completed within a week from the start of the procedure. On day 1, we assessed post-stroke fatigue, depressive symptoms, and functional outcomes. On day 2, participants performed a symptom-limited graded exercise test to determine the workload for their submaximal exercise test. On day 3, three repetitions of the submaximal constant-load exercise test were performed at 80% of the workload corresponding to the ventilatory threshold to assess the kinetics of cardiorespiratory variables^50^.

#### Assessments of post-stroke fatigue, depressive symptoms, and functional outcomes

Post-stroke fatigue was assessed using the 9-item FSS with each item rated on a 7-point Likert scale that ranged from 1 to 7 (1, strongly disagree; 7, strongly agree)^51^. The FSS score was calculated as the sum of the scores of the 9 items. A high score indicated a greater effect of fatigue on daily activities.

The 15-item Geriatric Depression Scale (GDS)^52^ was used to assess depressive symptoms. A GDS score of ≥ 5 denoted the presence of depressive symptoms. Motor function and independence in performing daily activities were assessed as functional outcomes. The total Stroke Impairment Assessment Set motor function score was measured to assess motor impairments in the paretic upper and lower extremities^53^. The Functional Independence Measure score was used to evaluate the degree of independence in activities of daily living^54^.

#### Exercise testing

Participants were instructed to refrain from food consumption for 3 h, caffeine intake for at least 6 h, and vigorous physical activity for 24 h prior to undertaking the symptom-limited graded and submaximal constant-load exercise tests^55^. The tests were performed on a recumbent cycle ergometer (Strength Ergo 240; Mitsubishi Electric Engineering Co., Ltd., Tokyo, Japan) that could be precisely load-controlled (coefficient of variation, 5%) over a wide range of pedalling resistance (0–400 W). Participants were instructed to maintain a target cadence of 50 rpm in all exercise phases^55^. Expired gas was measured on a breath-by-breath basis during the exercise test using an expired gas analyser (Aerosonic AT-1100; ANIMA Corp., Tokyo, Japan). Before data collection, the analyser was calibrated using gas mixtures with accurately known concentrations of oxygen and carbon dioxide. CO was measured on a beat-by-beat basis using a non-invasive impedance cardiography device (Task Force Monitor model 3040i; CN Systems Medizintechnik GmbH., Graz, Austria), as previously described^56^. Three short band electrodes, one on the neck and two below the thorax, were placed on the participants. Stroke volume was calculated using the following equation:

1 $${\text{Stroke }}\,{\text{volume }} = {\text{V}}_{{{\text{th}}}} \times {\text{LVET}} \times \left( {{\text{dZ}}/{\text{dt}}} \right)_{{{\text{max}}}} /{\text{Z}}_{0} ,$$

where V_th_ is the electrical participating thoracic volume, LVET is the left ventricular ejection time, (dZ/dt)_max_ is the maximal rate of decrease in impedance for a given heartbeat, and Z_0_ is the base impedance. CO was calculated as the product of stroke volume and heart rate. Impedance cardiography is a valid and reliable method for measuring cardiac haemodynamics at rest and during exercise ^56^. The measured values of cardiorespiratory variables were interpolated to 1 s intervals, time-aligned, and averaged into 5 s bins to derive the AVO_2_diff on a second-by-second basis ^13,24^ calculated based on Fick’s equation ^57^, as follows:

2 $${\text{AVO}}_{{\text{2}}} {\text{diff}} = \dot{V}{\text{O}}_{{\text{2}}} /{\text{ CO}}{\text{.}}$$

All participants rested for 5 min before taking the tests. The symptom-limited graded exercise test started with a warm-up at 0 W for 3 min followed by a 10 W increment every minute^55^. The test was terminated if the participant showed signs of angina, dyspnoea, inability to maintain a cycling cadence of > 40 rpm, hypertension (> 250 mmHg systolic or > 115 mmHg diastolic pressure), or a drop in systolic blood pressure of > 10 mmHg, despite an increase in workload^24^. To identify whether the maximal effort was reached during the exercise test, at least one of the following criteria had to be met: a < 150 mL min^−1^ increase in $\dot{\mathrm{V}}$O_2_ for > 1 min despite increased work rate, respiratory exchange ratio of > 1.10, or heart rate that was 85% of the age-predicted maximal heart rate calculated as 220 minus age^12^. $\dot{\mathrm{V}}$O_2_, CO, AVO_2_diff, $\dot{\mathrm{V}}$E, and the respiratory exchange ratio at peak exercise were defined as the average values obtained during the last 30 s of the exercise test^55^. In addition, the OUES was determined through calculating the slope of the regression line between $\dot{\mathrm{V}}$O_2_ and the log transformation of $\dot{\mathrm{V}}$E during the whole exercise period using the following equation:

3 $$\dot{V}{\text{O}}_{{\text{2}}} = a{\text{log}}\dot{V}{\text{E }} + b,$$

where the constant *a* is the OUES^58^. A low OUES represents a high amount of ventilation required in response to a given oxygen uptake, which indicates ventilatory inefficiency during exercise.

The ventilatory threshold was determined using a combination of the following criteria: the point where the ventilatory equivalent of oxygen reaches its minimum or starts to increase, without an increase in the ventilatory equivalent of carbon dioxide; the point at which the end-tidal oxygen fraction reaches a minimum or starts to increase, without a decline in the end-tidal carbon dioxide fraction; and the point of deflection of carbon dioxide output versus $\dot{\mathrm{V}}$O_2_ (the V-slope method), as previously described^24,59^. $\dot{\mathrm{V}}$O_2_, CO, AVO_2_diff, $\dot{\mathrm{V}}$E, and the respiratory exchange ratio at the ventilatory threshold were obtained.

The submaximal constant-load exercise test started with resting on the cycle ergometer for 3 min, followed by performing the exercise at 80% of the workload corresponding to the ventilatory threshold for 6 min^16,34^. The protocol was repeated three times, with a rest between each repetition. Data concerning the kinetics of the $\dot{\mathrm{V}}$O_2_, CO, AVO_2_diff, and $\dot{\mathrm{V}}$E at exercise onset were obtained through averaging the three repeats. Additionally, before modelling, we eliminated the first 20 s of data after exercise onset because the increase in $\dot{\mathrm{V}}$O_2_ during this period reflects merely an increase in the pulmonary blood flow rather than changes in tissue gas exchange^15^. To calculate the time constants of $\dot{\mathrm{V}}$O_2_, CO, AVO_2_diff, and $\dot{\mathrm{V}}$E at exercise onset, a non-linear least squares regression procedure (GraphPad Prism version 7.00 for Windows; GraphPad Software, CA, USA) was applied to the onset phase, using the following equation:

4 $${\text{Y }}\left( {\text{t}} \right){\text{ }} = {\text{ Y}}_{{{\text{baseline}}}} + {\text{ }}\left( {{\text{Y}}_{{{\text{steady}} - {\text{state}}}} - {\text{ Y}}_{{{\text{baseline}}}} } \right){\text{ }} \times {\text{ }}\left( {{\text{1 }} - {\text{ exp}}^{{ - \left( {{\text{t }} - {\text{ TD}}} \right)/{\text{ }}\tau }} } \right),$$

where Y (t) represents $\dot{\mathrm{V}}$O_2_, CO, AVO_2_diff, or $\dot{\mathrm{V}}$E at a given time (t); TD is the time delay; and τ is the time constant. Y_baseline_ and Y_steady-state_ are the average values of Y during the last minute of the resting period and exercise, respectively. Fit quality was determined using a coefficient of determination. The fitting procedure was considered acceptable if the coefficient of determination was > 0.85^33^. In addition, the ratio of τCO to τ$\dot{\mathrm{V}}$O_2_ > 1.00 indicated a slow increase in CO relative to $\dot{\mathrm{V}}$O_2_ at exercise onset.

### Statistical analyses

The sample size for examining the correlations between the FSS score and cardiorespiratory variables was computed at alpha = 0.05 and power = 0.80 using G Power software version 3.1.9.2 (Heinrich Heine University, Dusseldorf, Germany). Tseng et al.^18^ reported that the FSS score significantly correlated with the GDS score (r = 0.639) but not with $\dot{\mathrm{V}}$O_2_ at peak exercise (r = − 0.125) in 21 people with chronic stroke. Therefore, we calculated the required sample size to detect only a large effect size for correlation (0.50). Consequently, a minimum sample size of 26 participants was required. Assuming that 10% of the participants could be excluded, we aimed to recruit 30 participants.

The results are presented as medians (interquartile ranges) or means ± SDs. We examined the associations between the FSS score and cardiorespiratory variables using the Spearman’s rank correlation coefficient. To identify the potential confounding variables, we also determined the associations of participant’s characteristics with the FSS score and cardiorespiratory fitness variables that significantly correlated with the FSS score using the Pearson’s product-moment correlation coefficient, Spearman’s rank correlation coefficient, and unpaired t-test based on variable types. Additionally, we performed multiple regression analysis with forced entry to confirm whether the associations between the FSS score and cardiorespiratory fitness variables observed in the correlation analysis remained significant, even when adjusting for potential confounding variables. Statistical analyses were performed using Statistical Package for the Social Sciences software version 27.0 (International Business Machines Corp., NY, USA). Poisson (p) values < 0.05 were considered statistically significant.

## Supplementary Information

Supplementary Information.

## Data Availability

The datasets generated and/or analysed during the current study are available from the corresponding author on reasonable request.
