# Supplementary material for: Cardiorespiratory responses to exercise related to post-stroke fatigue severity
Source: Sci Rep. 2021 Jun 17;11:12780. doi: 10.1038/s41598-021-92127-w (PMC8211681; doi:10.1038/s41598-021-92127-w)
Supplement: Supplementary file 1 — Supplementary Information. [file 41598_2021_92127_MOESM1_ESM.pdf]

Supplementary Information

Cardiorespiratory responses to exercise related to post-stroke fatigue severity

Kazuaki Oyake, Yasuto Baba, Yuki Suda, Jun Murayama, Ayumi Mochida,

Yuki Ito, Honoka Abe, Kunitsugu Kondo, Yohei Otaka, Kimito Momose

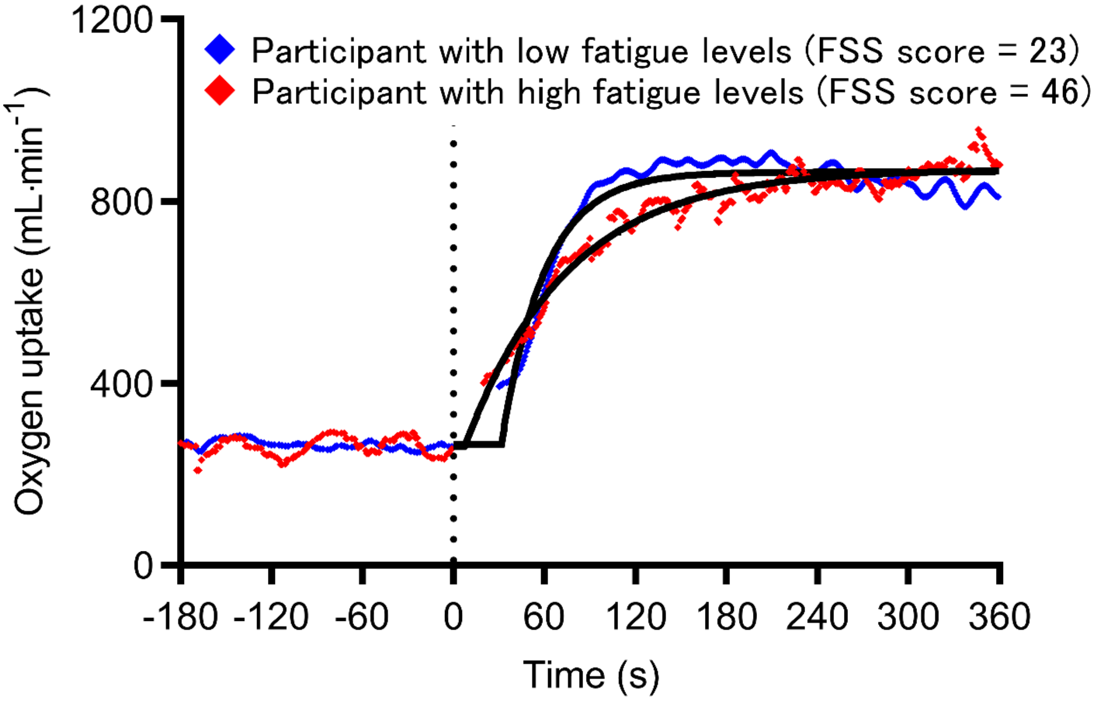

Supplementary Figure S1: Changes in oxygen consumption at the onset of exercise in a participant with low fatigue levels and one with high fatigue levels. Model fits on the data are displayed with solid lines. The vertical dashed line indicates the onset of exercise. The time constant and coefficient of determination of oxygen uptake kinetics in the participant with low fatigue levels (◆) were 28.4 s and 0.99, respectively. The time constant and coefficient of determination of oxygen uptake kinetics in the participant with high fatigue levels (◆) were 67.8 s and 0.99, respectively. FSS, Fatigue Severity Scale.

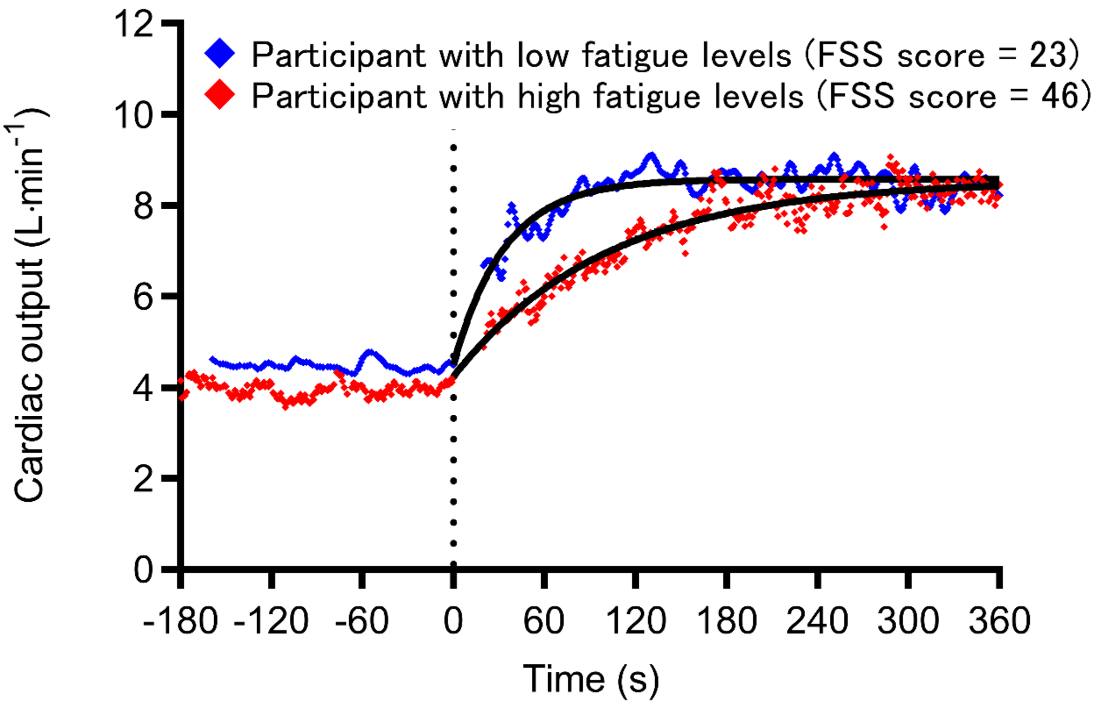

Supplementary Figure S2: Changes in cardiac output at the onset of exercise in a participant with low fatigue levels and one with high fatigue levels. Model fits on the data are displayed with solid lines. The vertical dashed line indicates the onset of exercise. The time constant and coefficient of determination of cardiac output kinetics in the participant with low fatigue levels (◆) were 33.3 s and 0.96, respectively. The time constant and coefficient of determination of cardiac output kinetics in the participant with high fatigue levels (◆) were 91.6 s and 0.98, respectively. FSS, Fatigue Severity Scale.

Supplementary Table S1: Multiple regression analysis to examine the association between the FSS score and $\tau\dot{V}O_2$, adjusted for logical confounding variables

Variable	β	t value	p value
Age-adjusted model: F (2, 20) = 3.002			0.072
$\tau\dot{V}O_2$	0.484	2.373	0.028
Age	−0.251	−1.234	0.232
Sex-adjusted model: F (2, 20) = 4.597			0.023
$\tau\dot{V}O_2$	0.428	2.313	0.031
Sex (male =1, female = 0)	−0.378	−2.040	0.055
Type of stroke-adjusted model: F (2, 20) = 3.754			0.041

$\tau\dot{V}O_2$	0.490	2.502	0.021
Type of stroke (ischaemic =1, haemorrhagic = 0)	−0.326	−1.663	0.112
Time since stroke-adjusted model: F (2, 20) = 2.095			0.149
$\tau\dot{V}O_2$	0.415	2.041	0.055
Time since stroke	−0.029	−0.144	0.887
Presence of depressive symptoms-adjusted model: F (2, 20) = 2.469			0.110
$\tau\dot{V}O_2$	0.418	2.087	0.050
Depressive symptoms (present = 1, absent = 0)	0.160	0.799	0.434
Functional Independence Measure motor score-adjusted model: F (2, 20) = 2.766			0.087

$\tau\dot{V}O_2$	0.441	2.213	0.039
Functional Independence Measure motor score	−0.212	−1.064	0.300

FSS, Fatigue Severity Scale; $\tau\dot{V}O_2$, time constant of oxygen uptake kinetics; β, standard coefficient.
